# The Impact of Unsupportive Social Support on the Injured Self in Breast Cancer Patients

**DOI:** 10.3389/fpsyg.2021.722211

**Published:** 2021-09-20

**Authors:** Valeria Sebri, Davide Mazzoni, Stefano Triberti, Gabriella Pravettoni

**Affiliations:** ^1^Department of Oncology and Hemato-Oncology, University of Milan, Milan, Italy; ^2^Applied Research Division for Cognitive and Psychological Science, IEO European Institute of Oncology IRCCS, Milan, Italy

**Keywords:** breast cancer, social support, Injured Self, patient, caregiver

## Introduction

A certain amount of social support is a fundamental human need and is associated with the belief that others have positive views about ourselves. Patients with chronic illness who receive high social support manage their health better (Nausheen et al., 2009; Martos-Méndez, 2015). For this reason, social support has been studied extensively in psycho-oncology. In breast cancer specifically, social support is crucial for the illness adjustment by decreasing distress, depression, and lowering the risk of recurrence (Drageset et al., 2016).

However, not all forms of social support that patients may receive from relatives and friends is beneficial (Breuer et al., 2017). Indeed, literature indicated that social support can be divided into positive support and negative support. Positive support, conceived as interactions that promote affection, has been extensively studied in patients with cancer (Ahn et al., 2017). On the other hand, negative support or when the recipient of support perceives it as unhelpful or feel social constraints by others received less attention from scholars (Breuer et al., 2017).

Integrating the social-health psychology perspective (Taylor, 2011) and the current directions in psycho-oncology that evidence the influence of self-representations on illness management (Ahles and Hurria, 2018; Sebri et al., 2020), in this opinion article we argue that unsupportive social relationships negatively affect one's self-representation, an important issue for health management in breast cancer patients (“Injured Self”). For example, Nieto et al. (2019) demonstrated that self-defining memories of patients can be associated with the experience of cancer. Before describing the specific routes through which the provision of social support may exert negative effects, we review the concepts of social support, unsupportive social relationships, and Injured Self in breast cancer patients.

### Definition and Types of Social Support

Social support may be defined as the experience of being loved and cared for by others, esteemed and valued, and part of a social network (Wills, 1991). Suurmeijer et al. (1995) identified four main types of social support: social-emotional or relationships rich in affection and companionship; instrumental, which is based on advice and practical suggestions; problem-oriented or actual supportive interaction focused on the resolution of a specific problem; and daily support, which relates to full-time assistance for both instrumental and socio-emotional means.

Furthermore, it should be made a distinction between perceived and received social support. According to life-span approaches (Uchino, 2009), pre-existing familial environment (e.g., family affection, which contributes to build a stronger Self able to face illness with psychological skills, such as proactive coping) influence perceived social support. This way, perceived social support has been linked to positive health outcomes. On the contrary, the current social/relational network affects received social support, which is situational in nature and therefore more volatile and less grounded in relationships and related social self-representations that developed and cemented over time.

### The Influence of Social Contexts

In line with the buffering model, which is defined as a process of support that protects individuals from potentially adverse effects of stressful events (Cohen and Wills, 1985), breast cancer patients are particularly susceptible to the availability of social support, at the time of diagnosis especially. Thus, social support can protect from feelings of helplessness and loss of self-esteem that arise from the perceived inability to cope with difficult situations. The perception that others will provide the resources that redefine the harmful events and bolster one's perceived ability to face imposed demands can attenuate distress thanks to the assurance of emotional and esteem support and a sense of security (Zhang et al., 2017). However, it is fundamental to consider perceived social support from a constructionist point of view as well (Sarason et al., 1986, 1991; Lakey et al., 1992): multiple studies have demonstrated that social support is influenced by the recipient's perception of the others' evaluation on themselves and their personality characteristics, such as attachment style. For example, individual with insecure attachment style may perceive supportive messages as patronizing, insensitive while secure people benefit from them (Collins and Feeney, 2004). Also the socio-cognitive theory highlights that perceived social support is influenced by characteristics of receiver, supporter, and their relationship (Haber et al., 2007). Furthermore, factors associated with the larger social and cultural context of supportive interactions may affect the perception of support (Badr et al., 2001) (e.g., more restrictive cultures may limit one's possibility to develop supportive relationships outside of the family nucleus).

### Unsupportive Social Relationships

Some types of social support may negatively affect breast cancer patients' quality of life (Shiozaki et al., 2011). A similar phenomenon has been described in several medical conditions in terms of “problematic support” (Revenson et al., 1991), “oppressive support” (Mazzoni and Cicognani, 2014; Mazzoni et al., 2017), and “negative support” (Shiozaki et al., 2011). Although patients may perceive the supportive intent of the provider, the social interaction could be perceived as detrimental and can lead to promote disengagement coping strategies (Nouman and Zanbar, 2020). Moreover, in the long term, studies evidenced that breast cancer patients who experience detrimental interactions showed high depressive symptoms and low physical well-being (Yu and Sherman, 2015). Similarly, avoidant communication about cancer issues generally increases anxiety, depression, and lower relationship satisfaction (Manne et al., 2006; Donovan-Kicken and Caughlin, 2011). This could be related to cognitive distortions typical of chronically ill patients, who tend to focus on disease-related information within the communication with doctors or caregivers, which possibly leads to catastrophizing and detrimental emotions (attentional and interpretation bias) (Savioni and Triberti, 2020), or are not able to express their actual emotional needs, which can make patient-caregiver relationship more difficult (Marzorati et al., 2018). In other words, unsupportive social support should not be understood simply as the lack of support, but as social interactions that do not meet the needs or cognitive representations of the patient. Is relevant to know that social support can be unintentionally negative because support providers do not even know what kind of actions are supportive. Thus, also providers may experience further stress that results in a vicious cycle of detrimental interactions (Shiozaki et al., 2011).

### Injured Self in Breast Cancer Patients

Recently we have proposed the term “Injured Self” to describe how experiencing a chronic illness may deeply affect one's own self-representation, both bodily and narrative (Brown et al., 1986; Sebri et al., 2020). According to Self discrepancy theory and related studies the self is a malleable, dynamic and multiple construct (Markus, 1977; Higgins et al., 1985; Markus and Kunda, 1986), multiple self-representations coexist (e.g., ideal self, actual self, ought self, good and bad self) and can be activated to guide behaviors at any particular moment due to a number of factors that make them salient in a social situation. Discrepancy between selves, as well as lack of integration in one self-representation, have been consistently linked to mental and physical health issues (Higgins et al., 1985; Mason et al., 2019; Triberti et al., 2019; Sebri et al., 2021a). The experience of chronic illness can affect the perception on both bodily and narrative self-representations Specifically, oncological treatments can alter patients' self-concept as they experience consequences (or effects) of treatments (such as hair loss, sexual disturbances or breast removal) (Clemmey and Nicassio, 1997; Sebri et al., 2021b). In this sense, patients can perceive their bodies as a source of danger and fear (Sebri et al., 2021b) and adopt compulsive “checking behaviors” (Butow et al., 2018). After the breast cancer experience, interoceptive sensations can become more and more salient, increasing survivors' attention to bodily signals that are possibly related to one's own health, for example touching sensitive areas of their breast looking for nodules (McGinty et al., 2016). This way, internal somatic cues and checking behaviors not only can be interpreted as continual reminders of the disease or indications that the disease has returned, but also as a deep alteration of the experience of the body that affect quality of life daily (De Vignemont, 2018).

At the same time, regarding narrative representation, one's identity can be affected by storylines articulated around the relationship between self-representations and autobiographical memories, such as the experience of a breast cancer (Nieto et al., 2019). This way, both bodily and narrative representations connected to the illness experience can concur to an added self-representation, defined as the Injured Self (Sebri et al., 2020). The Injured Self actively influences everyday life decisions because it is connected to the ongoing expression of one's own identity in emotions and behaviors (Giffard et al., 2013). The experience of a non-association between autobiographical memories, bodily sensations, and real-life events can destroy a coherent and integrated sense of Self (Sebri et al., 2020). Breast cancer patients have to reframe the overall Self with the integration of the Injured Self as that of a patient based on the autobiographical memory of the cancer experience, body image distortions, and their related emotions.

## Social Support and Injured Self

Different forms of positive social support may help breast cancer patients to integrate various self-schemas into a coherent one. For example, peers can promote group belonging aiming at overcoming patients' stigmatization (Sheikhpourkhani et al., 2018; Pardede et al., 2021); partner support is relevant to face sexual disturbances caused by changes in body image (Manne et al., 2005; Yu and Sherman, 2015). In general, being treated as a person, and not just as a patient, is essential to restore the overall Self and promote patient adherence to treatment (Schulman-Green et al., 2016). Breast cancer patients who perceive a sense of autonomy tend to be actively involved in a shared decision-making process becoming more able to adopt adaptive coping styles (Sebri et al., 2020). In the light of the benefits of a good quality social support for the patient's Self, it is of primary importance to identify the potentially risky provisions of support that may aliment specific Injured Self dimensions.

### The Impact of Problematic Social Support on Injured Self

Taking into account the four types of social support provision (social-emotional, instrumental, problem-oriented, and daily social support proposed by Suurmeijer et al. (1995), we explore which type of social support may further aggravate the Injured Self features and its consequences in breast cancer patients (see Table 1). We will also try to specify these mechanisms' sensitiveness to patients' individual expectations, needs and personal characteristics, as these are determinant in the final reception of social support (Collins and Feeney, 2004).

- Illness denial: family members can be highly burdened at the time of diagnosis and may avoid facing problems with the intention of keeping patients far from distress (Sauer et al., 2019). For example, both patients and caregivers may underestimate the effects of oncological treatments. Therefore, thoughts and sensations are suppressed (Shiozaki et al., 2011) and patients cannot handle their concerns directly. Thus, illness denial tend to promote an experience of patients' self-fragmentation characterized by low self-esteem as well as negative Body Image with difficulties in the integration of the Injured Self into the overall Self. Illness denial may protect patients from overwhelming experience of cancer diagnosis and its treatment in the short term, but in the long run they will cause damage by delaying or hindering health management;
- Taking over: when providers are overly involved in patients' affairs, they may only base their support on instrumental intervention aiming at solving patients' difficulties. The caregiver's excessive involvement may increase of patients' loss of control, inability to be self-sufficient and high vulnerability to life events as well as a lack of coping resources (Boutin-Foster, 2005). This is more likely to happen to patients in an emotional “blackout” after the diagnosis (Graffigna et al., 2016), who are more likely to passively accept caregivers' behaviors and possibly shape their understanding of the illness on them. As a consequence, breast cancer patients do not perceive control over their own social environment and may reject oncological treatments or accept them just passively, without shared decision-making;
- Poor communication with caregivers: couples find troubles communicating sensitively and openly regarding breast cancer topics for various reasons (Goldsmith and Miller, 2013). On one side, patients tend to protect themselves and others from the discomfort of discussing cancer-related issues, such as sexuality concerns and fears about disease progression and death (Yu and Sherman, 2015). On the other side, some women avoid open communication due to the experience of unsolicited information, unwelcome suggestions or critiques about how patients should cope (Boutin-Foster, 2005). The more people avoid talking about cancer-related issues, the fewer possibilities patients have to elaborate their illness, increasing distress, anxiety, and depression (Munro et al., 2014). This is the result of a disengaged type of instrumental support in which patients perceive not being worthy of attention in reference to their needs and desires;
- Illness-centered view: the attitude to interact just about the cancer experience may increase the stigma of being a patient everyday (Penner et al., 2018). On one side patients may need to be supported exactly in developing attentional focus on their disease, especially when in denial, which deserve to be understood and managed as a spectrum from beneficial coping to maladaptive self-harm with subconscious disavowal (Pene and Kissane, 2019). However, ultimately patient engagement requires the patient to recover a positive life projectuality beyond the disease in order to develop hope and intrinsic motivation (Graffigna et al., 2016). For example, not asking breast cancer patients what their dreams and future goals are may lead patients to perceive themselves as isolated from society with sensations of shame and guilt. Thus, patients feel different from others due to their illness. Moreover, the patients' stigmatization increases difficulties in intimacy and sexual life due to sensations of being worthless, defeminized, and sexually unacceptable (Cesnik et al., 2013).

**Table 1:** Four main unsupportive social support types that may promote Injured Self features and its emotional and behavioral consequences.

**Social support**		**Injured Self**	
**Type of social support**	**Problematic characteristics**	**The Injured Self features**	**Consequences**
Social-emotional social support	Illness denial	Worsening of self-fragmentation in those patients who are already adopting biased coping strategies	- Low self-efficacy and self-esteem - Negative body image - Difficulties in the integration of the Injured Self into the overall Self
Problem-oriented social support	Taking over	- Fear of not being always self-sufficient, without autonomy in patients with insecure attachment, dependent personality	- Accept/reject treatments passively - Perception of hopelessness and absence of coping resources
Instrumental social support	Poor communication with caregivers	- Perception of doing not be worthy of attention - High negative emotions	- High distress, anxiety, and depression - Avoidance of patients' needs and expectations - Suppression of negative feelings - Fewer possibilities to elaborate illness experience
Daily social support	Illness-centered view	Seeing oneself only as a patient, for those patients who need to recover self-projectuality, hope and intrinsic motivation	- Isolation - Sexual disturbances - Lack of future goals and dreams

Nowadays, literature shows the efficacy of support-expressive group therapy in improving emotional well-being in breast cancer (Bellver-Pérez et al., 2019). We argue to propose structured psychological interventions based on the training of positive social support to cope with Injured Self and prevent mental burden. For example, assessing the patient' consensus of receiving advice could be appreciated by patients who may tend to be actively involved in decision processes (Boutin-Foster, 2005); moreover, improvements in many coping behaviors may offer functional benefit in psychological adjustment (Donovan-Kicken and Caughlin, 2011). Additionally, the social network outside the family (e.g., online communities) may be functional to improve quality of life too (Sauer et al., 2019). As perceived social support is influenced by the recipient's characteristics and pre-existent context (Collins and Feeney, 2004), it could be important to profile patients in terms of needs and personality traits so to develop ad-hoc resources for optimizing social support strategies. This would allow health professionals to work in conjunction with caregivers to raise awareness on the functions of social support and to identify the best support conducts to enact toward the patient (Cherif et al., 2020).

## Conclusions

Problematic social support may contribute to one's self-fragmentation. In breast cancer patients, such a process is particularly evident, since women are affected by “injuries” to the self (Boutin-Foster, 2005). As aforementioned, not all types of support are effective. Patients differ in their beliefs and needs of being involved in the healthcare process. Future experimental research could better explore the relationship between social support and Injured Self features, personal needs and personality characteristics. Different types of social support could be measured by assessing their influences on patients' self-coherence and the subjective interpretation of the events connected to the Injured Self. For example, interviews might investigate how social support can affect patient and caregiver communication and women's adaptive coping skills. Finally, future investigations should still explore how to manage Injured Self through positive social support by examining the role of personality traits, stage of cancer and timing of treatment, and cultural background (Shiozaki et al., 2011). For instance, relationships differ in Western and Eastern cultures in terms of contextualism and emotional dependency (Ferdiana et al., 2018). Likewise, the primary and fundamental psychological needs associated with social support (Williams, 2009) should be taken into account to tailor a personalized psychological intervention on individuals' characteristics and needs aiming at managing Injured Self's characteristics. Such interventions should not be targeted on patients only, but their caregiving network(s) as well, in order to raise awareness on the opportunities and risks within social support and to promote the more effective support conducts. Taking into account life-span theories of perceived/received social support, future research and literature review may also explore how social support should adapt over different phases of breast cancer management trajectories.

## Author Contributions

VS and DM conceived the ideas presented in the article. VS wrote the first draft. ST contributed with discussion on the ideas presented. DM and ST edited the manuscript. GP contributed with important intellectual contents and supervised the whole process. All authors contributed to the article and approved the submitted version.

## Conflict of Interest

The authors declare that the research was conducted in the absence of any commercial or financial relationships that could be construed as a potential conflict of interest.

## Publisher's Note

All claims expressed in this article are solely those of the authors and do not necessarily represent those of their affiliated organizations, or those of the publisher, the editors and the reviewers. Any product that may be evaluated in this article, or claim that may be made by its manufacturer, is not guaranteed or endorsed by the publisher.
